# Exploring the context of sedentary behaviour in older adults (what, where, why, when and with whom)

**DOI:** 10.1186/s11556-015-0146-7

**Published:** 2015-10-07

**Authors:** Calum F. Leask, Juliet A. Harvey, Dawn A. Skelton, Sebastien FM Chastin

**Affiliations:** Institute of Applied Health Research, School of Health & Life Sciences, Glasgow Caledonian University, Glasgow, G4 0BA UK

**Keywords:** Camera, Seniors, Accelerometer, Inclinometer, Lifelogging, Sitting, SITONAUMY

## Abstract

**Background:**

Older adults are the most sedentary segment of the population. Little information is available about the context of sedentary behaviour to inform guidelines and intervention. There is a dearth of information about when, where to intervene and which specific behaviours intervention should target. The aim of this exploratory study was to obtain objective information about what older adults do when sedentary, where and when they are sedentary and in what social context.

**Methods:**

The study was a cross-sectional data collection. Older adults (Mean age = 73.25, SD ± 5.48, median = 72, IQR = 11) volunteers wore activPAL monitors and a Vicon Revue timelapse camera between 1 and 7 days. Periods of sedentary behaviour were identified using the activPAL and the context extracted from the pictures taken during these periods. Analysis of context was conducted using the Sedentary Behaviour International Taxonomy classification system.

**Results:**

In total, 52 days from 36 participants were available for analysis. Participants spent 70.1 % of sedentary time at home, 56.9 % of sedentary time on their own and 46.8 % occurred in the afternoon. Seated social activities were infrequent (6.9 % of sedentary bouts) but prolonged (18 % of sedentary time). Participants appeared to frequently have vacant sitting time (41 % of non-screen sedentary time) and screen sitting was prevalent (36 % of total sedentary time).

**Conclusions:**

This study provides valuable information to inform future interventions to reduce sedentary behaviour. Interventions should consider targeting the home environment and focus on the afternoon sitting time, though this needs confirmation in a larger study. Tackling social isolation may also be a target to reduce sedentary time.

## Background

Promoting active ageing to decrease the burden of chronic disease and increase quality of life in later life has become a key public health focus [1]. Despite the well documented benefits of physical activity for health, inactivity is at pandemic level [2]. The most prevalent form of inactivity is behaviours where sitting or lying is the dominant mode of posture and energy expenditure is low. These behaviours have recently been clustered under the single term of sedentary behaviour (SB) [3].

Older adults are the most sedentary segment of society, with 2 of 3 spending over 8.5 h per day in sedentary activities [4, 5]. Too much time spent sedentary is associated with poorer health outcomes, less successful ageing [6, 7] and premature mortality [8]. National and international guidelines for physical activity now include specific recommendations to reduce SB for older adults [9, 10]. Therefore, interventions are needed to decrease SB in older adults. According to health behaviour theories, for example the duel process theory [11] and socio-ecological model [12], behaviour and individual choices are determined by social and physical environmental context. The NIH stated it is crucial to measure and understand the context of SB in order to develop effective intervention and inform public policy [13]. The context of SB is defined by the SITAUNOMY consensus taxonomy as the specific sedentary activity (what), its purpose (why), the location (where), time (when) and social setting (with whom) in which it occurs [14].

Currently there is a dearth of information about the context of SB in older adults. There are no objective data about what type of SB older adult engage in and for what purpose (e.g. rest, transportation, leisure). This is important as they might not be all as modifiable or have the same health effects [15]. Similarly, there is no objective information to guide where and when to intervene such as whether SB occurs outdoors or indoor (at home or in community facilities), at what time of the day and whether this is solitary or social time.

Novel life-logging technology combining activity monitors and body worn timelapse photography cameras enables continual tracking of individuals’ surroundings from their visual perspective. Currently this provides the best solution to obtain objective data about the context of SB. This technology has been used successfully to determine the context of physical activity in adults [16] and was found to be an acceptable technology by older adults [17].

The aim of this exploratory study was to quantify objectively what type of SB older adults engage in and when, where, why and with whom these occur.

## Methods

**Fig. 1:**
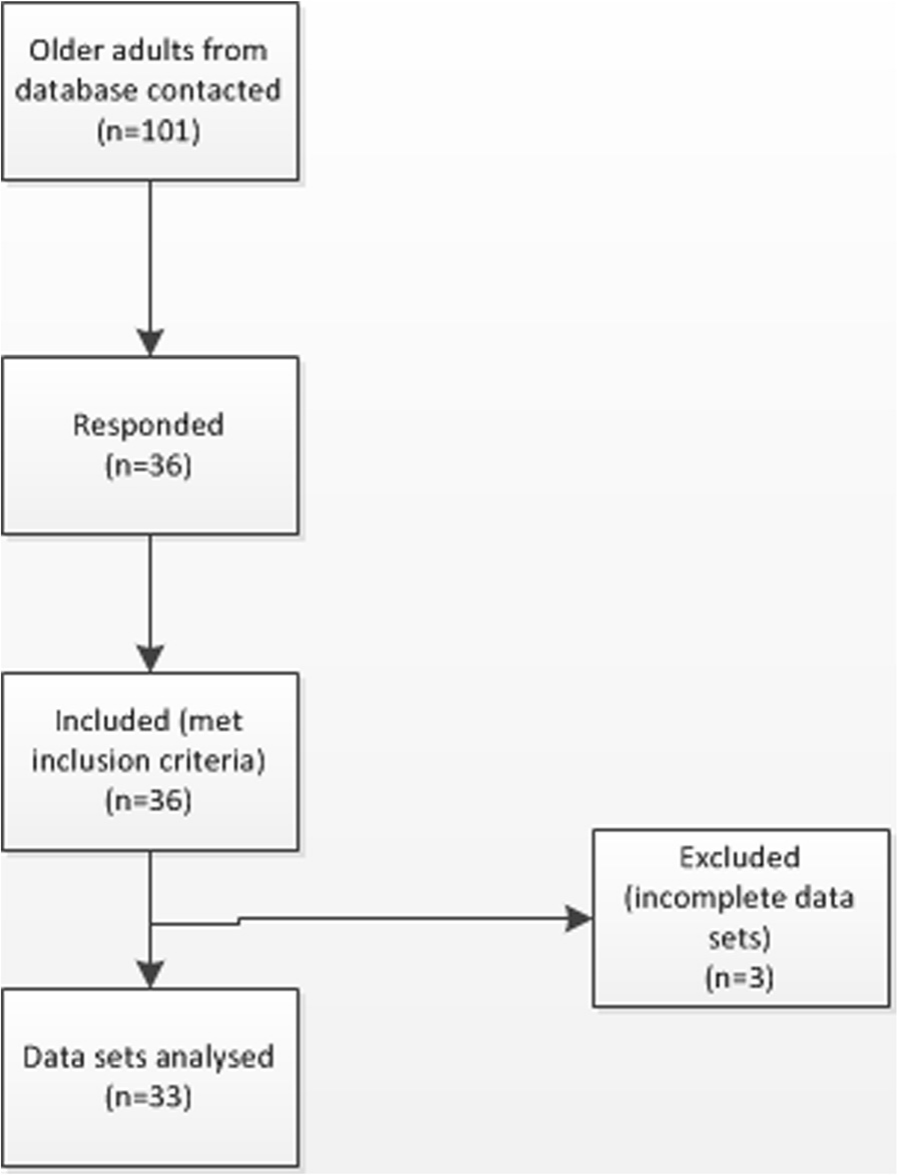
Inclusion criteria for participation

This cross-sectional study consisted of a convenience sample of 36 community dwelling, medically stable older adults aged over 65 years, recruited from the Glasgow Caledonian University Older Adult Volunteer Research Database. The Older Adult Volunteer Research Database contains [101, as of July 2015] such individuals with a wide variety of controlled medical conditions whom have consented to being approached for research studies. No one on this database has any acute or uncontrolled conditions.^1^ All the older adults in the database were contacted via postal letter with the study information. 40 participants were aimed to be recruited and 36 individuals responded to the invite. This was considered enough to give a spread of experiences in this novel approach to considering sedentary behaviour and context. Volunteers who were willing to participate sent back a signed consent form. Inclusion and exclusion criteria were: 65+ years of age; community dwelling; able to ambulate independently; able to give informed consent; no allergy to adhesive tape (necessary for securing activPAL). All 36 responders met the inclusion criteria (see Fig. 1).

**Fig. 2:**
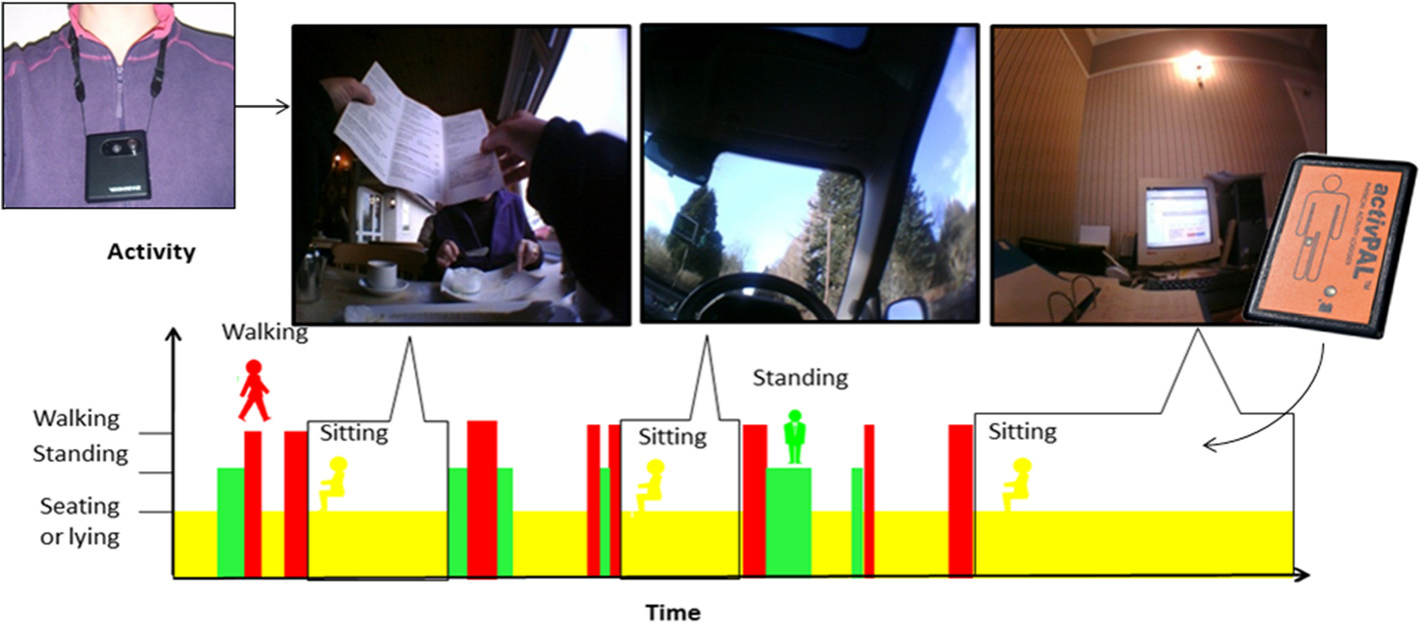
The concept of using activity monitoring and images in combination

The system used in the study comprised of a timelapse camera (Vicon Revue™ formerly known as SenseCam) and an activity monitor (activPAL™) as illustrated in Fig. 2. The output of the activity monitor enabled identification of the start and duration of SB periods and extracted the relevant pictures from the timelapse camera data (see Fig. 2). The activPAL™ is a thigh mounted activity monitor of objectively measured free-living activity. The monitor has been shown to be accurate for measurement of static and dynamic activity, and posture, and is regarded as a gold standard for detecting periods of SB [18]. Vicon Revue™ is a body worn timelapse camera, which passively records the context of the participant’s free-living behaviour by collecting images when a movement or a change in environment is detected, for example temperature or light. On average, it collects 5 pictures per minute and the validity of using timelapse cameras and accelerometers in tandem has previously been shown [19].

The participants were met at their preferred location, where they received detailed instruction about how to use the equipment and provided with a manual. They were requested to wear the equipment continuously, excluding water based activities and times were it was inappropriate to wear a camera (e.g. school, toilets), for seven days. On a second visit to collect the devices, participants were specifically asked if they had altered their behaviour whilst wearing the equipment.

The output of activPAL was used to identify the start and duration of SB periods. Pictures taken by the timelapse camera corresponding to these periods were extracted. Pictures for each bout of SB longer than 2 minutes were reviewed by two independent researchers and classified using the Sedentary Behaviour International Taxonomy (SITONAUMY) [14]. When the independent researchers disagreed, a third member of the research team was involved in the discussion until a resolution was agreed. Each sedentary bout was characterised by 9 independent facets covering contextual components including the purpose, type, time, environmental and social context of sedentary bouts. If the context of SB could not be determined from the picture, the bout was classified as undetermined. Simple descriptive statistics were computed in terms of time spent in each category and their frequency in these 9 independent facets to characterise the context of SB in older adults. Sedentary bouts were classified as a percentage of the waking day. Sleep diaries were not kept, therefore waking day was taken from the first point in the morning where there was obvious movement to when movement stopped late in the evening. No activity overnight was considered in the data. Sleep time during the day could not be determined. A minimum wear time of one day was considered acceptable for data collection 1) a reliability issue for the timelapse camera over a period of several days was noted due to the camera requiring a daily recharge 2) Nicolai [20] previously suggested that one day of data may provide sufficient information.

Ethical approval was gained from Glasgow Caledonian University ethics committee for the study. All participants received study information packs and signed an informed consent form before being recruited in the study. All operation of the timelapse camera, including data handling, adhered to the best practice guidelines for using this technology [21, 22]. All participants consented to taking part in the study and to the anonymised images being used for the study, in peer reviewed journals and presentations.

## Results

Out of a total of 36 participants (M = 13, F = 23), 33 were included for analysis, as 3 did not have image data due to the camera malfunctioning. Charging issues with the camera meant that most participants were only able to collect one day of camera data. 52 days of data were provided with a minimum and maximum of 1–7 days being collected. The median number of days of device wear was 1 day, with the average wear time being 1.5 days. As Marshall et al. [23] has previously reported no significant difference between weekday and weekend sedentary behaviour in older adults, this information was not collected. The age of the participants ranged from 65 to 82 with a median of 73.3 years. They had an average BMI of 25.6 (±5.2) kg/cm^2^, 43.3 % of the participants were married and 33.3 % widowed. Based on the Scottish Index of Multiple deprivation [24], the social economic distribution of the participants was 10, 23.3, 20.0, 26.7, 20 % from the least to most deprived quintile. On average, per day, participants engaged in 30 (range 11 to 35) bouts of SB longer than 2 min and spent on average 59.2 % (range 28.3 to 94 %) sedentary. No participant felt the wearing of the devices altered their normal behaviour.

The context of SB is presented as pie charts as percentage of bouts per day and the percentage of time per day spent in each relevant facet of the SITONAUMY classification system (type, purpose, time, environment context, social context and associated behaviour) of sedentary bouts.

**Fig. 3:**
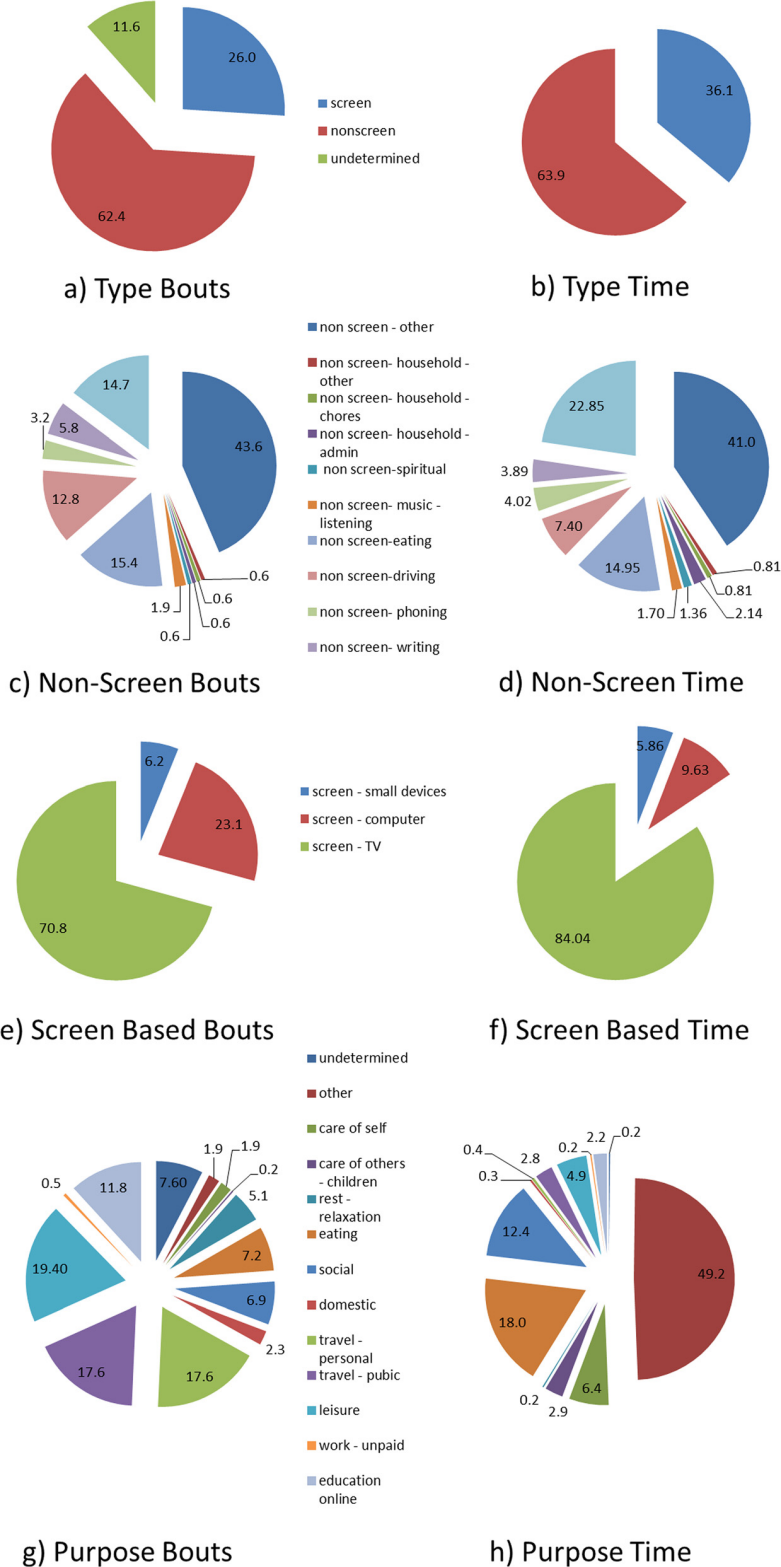
Distribution of the type of sedentary bouts **a**) and time **b**). Distribution of non-screen based sedentary bouts **c**) and **d**) (% of day). Distribution of the screen-based sedentary bouts **e**) and time **f**) (% of day). Distribution of the purpose of sedentary bouts **g**) and time **h**) (% of day)

Figure 3 shows the distribution of the type of sedentary behaviour through the day in term of percentage of the number of sedentary bouts and total sedentary time. Non-screen activities (62.4 %) were most frequent, screen-based activities represented 26 % of bouts and 11.6 % of bouts were undetermined. The average time in non-screen activities accounted for 63.9 % and screen activities for 36.1 % of time.

Examining these non-screen based categories in more detail, driving, reading and eating contributed to 12.8, 14.7 and 15.4 % of the total sedentary bouts (see Fig. 3c). This corresponded to 7.4, 22.9 and 7.4 % of total sedentary time (see Fig. 3d). The majority of non-screen based sedentary bouts and time spent sedentary could not be ascribed to specific behaviours and therefore classed as other (43.6 % of bouts and 41 % of time).

Screen-based sedentary activities, for example screen – TV, screen – computer and screen – small devices were responsible for 70.8, 23.1 and 6.2 % of total sedentary bouts (see Fig. 3f). These accounted for 84, 9.6 and 5.9 % of total sedentary time respectively (see Fig. 3e). Screen – other (0.5 %) was attributed to the remainder of time.

In the purpose category, leisure was responsible for 19.4 % of the sedentary bouts, corresponding to 49.2 % of the average time (Fig. 3g and h). Social (18 %) and eating (12.4 %) were the other dominant sedentary time purposes, corresponding to 6.9 and 7.2 % of bouts respectively. Despite travel by public or personal means being dominant in terms of sedentary bouts (17.6 % each), they accounted for only 6.4 and 2.9 % of the total sedentary time respectively.

**Fig. 4:**
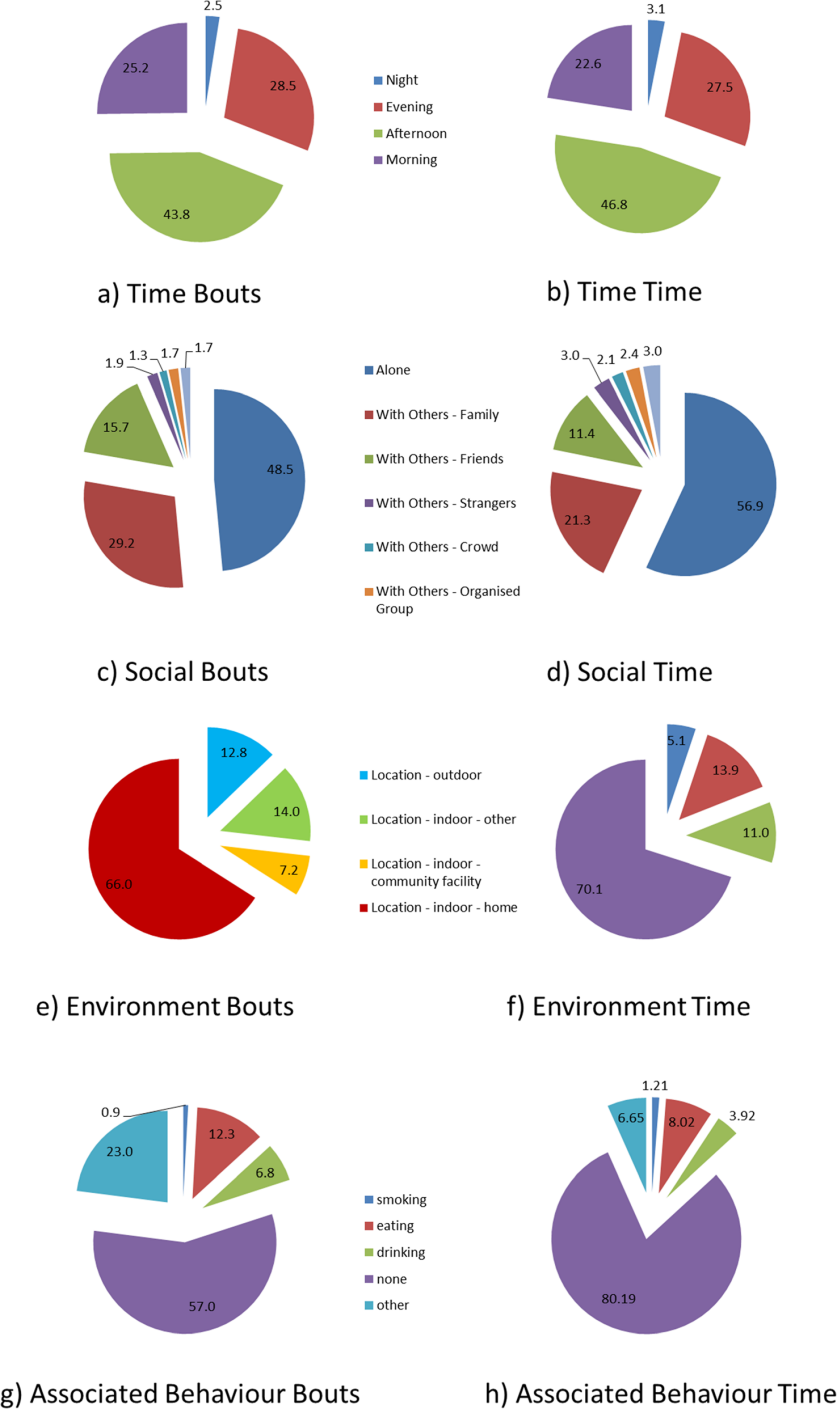
Distribution of sedentary bouts **a**) and time **b**) throughout the day (Morning: 0700-1159-1200, Afternoon: 1200–1659, Evening: 1700–1959, Night: 2000–0659). Distribution of the social context of sedentary bouts **c**) and time **d**) (% of day). Distribution of the environmental context of sedentary bouts **e**) and time **f**) (% of day). Distribution of associated behaviour with sedentary bouts **g**) and time **h**)

The majority of sedentary bouts occurred in the afternoon (43.8 %), with evenings (28.5 %) and morning (25.2 %) accounting for almost a quarter of the total percentage each (Fig. 4a). This corresponded to 46.8, 27.5 and 22.6 % of total sedentary time respectively (Fig. 4b).

Participants predominately were sedentary on their own (56.9 %). Time spent with family members (21.3 %) and friends (11.4 %, Fig. 4d) were the other two dominant social contexts of sedentary time. This corresponded to 48.5, 29.2 and 15.7 % of bouts respectively (see Fig. 4c).

Most sedentary bouts were indoors (94.9 %) with 5.1 % occurring outdoors (see Fig. 4e). This corresponded to 87.2 % of time indoor and 12.8 % of time outdoor (see Fig. 4f). 70.1 % of sedentary time was in the Participants’ home and 11 % occurred in indoor community facilities.

Predominately, sedentary time did not occur alongside associated health behaviours (80.2 % of the time and 57 % of bouts). Eating (8 %) was the most prevalent associated behaviour, accounting for 8 % of time and 12.3 % of the bouts (see Fig. 4g). Drinking was the next most frequent and prevalent associated behaviour (3.9 % of time and 6.8 % of bouts).

## Discussion

This is the first study to quantify objectively when, where, why, with whom and for what purpose older adult engage in SB. Some findings were as predicted in Owen’s ecological model [12]. Notably, household, leisure and transport were all identified as key domains where prolonged sedentary activities are likely to occur. Here, participants were found to be sedentary in their own home for 70.1 % of the time. Transport, both personal and public, accounted for 35.2 % of sedentary bouts, but bouts were generally not prolonged.

Leisure was the most frequent purpose of SB and was responsible for 49.2 % of sedentary time. Leisure SB mostly occurred when individuals were alone (56.9 % of time) and this might be a potential factor contributing to the association of poor health with prolonged SB. Loneliness is a predictor of impaired cognition [25] and all-cause mortality [26] in older adults, with sedentary time also shown to be associated with poor mood [27]. It is therefore important to examine whether loneliness is a determinant towards SB, or vice versa. An association between loneliness and increased risk of sedentary time was acknowledged by Netz et al. [28], whilst Lauder et al. [29] discussed that lonely individuals had a decreased belief that being physically active was a desirable behaviour. Combined, findings suggest that loneliness may increase an individual’s risk of being sedentary and this may be an important segment of the older adult population to target when implementing interventions to reduce sedentary time.

Conversely, time with others in social activities made up 6.9 % of bouts but 18 % of time, an infrequent but prolonged sedentary activity. It could be questioned if this social time is detrimental to health even if it is predominantly sedentary. It has been suggested that social isolation may influence depression in older male adults [30], therefore implying that social activities have a mental health benefit. While the majority of SB appears totally passive, older adults also spent a sizable amount of their SB in either social or cognitively demanding activities such as reading (22.9 % of non-screen SB). These engaging activities facilitate cognitive function, especially in old age [15], therefore it is worth asking whether future interventions to reduce SB should target all sedentary periods or focus on cognitively passive and socially isolated periods. Examples of this may include time doing nothing and screen time, both of which were dominant behaviours. Screen time, which occurred in 26 % of sedentary bouts, has been found to be associated with loneliness and depression [31].

The fourth domain in Owen’s [12] ecological model is occupation; however paid work is not relevant to the majority of the older population. Instead, care of others was responsible for 5 % of sedentary time; therefore caring and volunteering may be the occupation domain in this population. It would appear that these older adults used caring for others as an activity to reduce their sedentary time, as opposed to increase it [32]. Caring for others maintains a sense of a societal role [32] and gives more personal relevance and meaning to their daily activities. Additionally, associated behaviours which may be considered detrimental to health, for example smoking and drinking, were found to be limited in this sample (80.19 % of sedentary time had no corresponding associated behaviour). This does not reflect previous research to suggest a link between obesity and sedentary time, for example due to snacking whilst watching TV [33].

The study does have some limitations. The sample considered is small and while it includes a wide range of age, social economic and health status and physical capacity, it is not fully representative of all older adults. It is likely, due to the convenience sampling that there was selection bias to those interested in their sedentary behaviour context and therefore likely to be less sedentary than the general population. As such, the results might not be generalisable to the whole population. However, some of the results can be compared to that of large scale studies and representative surveys. Older adults had an average BMI of 25.6 and similar to the Scottish Health Survey [34], were classed as overweight. Here, older adults were found to spend an average of 30 minutes daily on the computer. Harvey et al. [4] previously reported a weighted mean of 21.5 min per day of computer use in a systematic review of older adult sedentary behaviour, reinforcing the validity of the results. There was, however, a larger difference visible in TV viewing time here (4.3 h) compared to a weighted mean of 3.3 h found by Harvey et al. [4], however previous work has used self-report methods which may decrease reliability. Overall, sedentary time here was measured as an average of 14.2 h per day. This compares well to accelerometry based work by Healy et al. [35] who found individuals to be sedentary for 13.7 h daily. This suggests that despite the small and convenience sample nature of this study, it captured reasonable estimates of the context of SB in older adults.

A percentage of sedentary bouts could not be classified. Firstly, in some instances, no images were captured because the camera was covered, or the participant pressed the privacy button or the camera malfunctioned. This resulted in 7.6 % of sedentary bouts having an undetermined purpose. However, this corresponds to only 0.4 % of participants’ total sedentary time, showing that this missing data would not significantly change the findings presented.

Although participants said wearing the camera did not affect their behaviour, it may have done. Indeed it is well known that bodyworn sensors tend to have some reactivity [36]. In the future this should be investigated and studies should consider using this technology over longer period to normalise behaviour.

The method used in this study can provide objective assessment of the context but is relatively costly to deploy. It generates large amount of data that require secure storage and intensive data processing and coding, each individuals’ data processing taking more than 2 h. For large scale studies and population surveillance self-reported measures might be more pragmatic [37]. However, the development of such measure should be informed by studies based on objective measure such as this one to ensure that the self-reported tools capture the most important and prevalent context.

The main strength of this study is the use of objective measures combined with the SITONAUMY classification systems. The objective measures mean that no prior assumptions were made about what the context of SB in older adults is. In addition, the SITONAUMY classification system used has two major benefits. First it minimised any potential overlap between categories which a common problem faced by studies investigating context of behaviour [34]. Second, it is not based on a single ontology but on a multidisciplinary consensus which increases the validity of the classification.

## Conclusions

This is the first study which measured objectively the context of SB in older adults. The results indicate that the majority of sedentary time is accumulated at home, in the afternoon, for leisure and in social isolation. These results ideally should be confirmed in a larger sample representative of the older population, for example random sampling from GP practices. Interventions wishing to reduce sedentary time in this population should consider targeting these four factors. The loneliness experienced during SB might in part contribute to the negative association between SB and health. The majority of the SB leisure time is spent in front of screens (TV, computer) however SB is also prominent during social time and during cognitively challenging activities which might have positive mental health effects.

## Footnotes

1 Recruitment for the older adult database occurs throughout the year at engagement events with local older adult community groups, for example U3A and lunch clubs. Participants consent to being contacted for any research studies within the department that need over 65’s. They complete a health questionnaire at time of consent to allow purposeful sampling. They need to be community dwelling, medically stable and able to attend the university for the research. For each research project, volunteers are contact via mail with a description of the research study and provided with return envelopes. Volunteers who agree to take part are then sent participant information sheets and consent forms.
